# The Structure of an Injectisome Export Gate Demonstrates Conservation of Architecture in the Core Export Gate between Flagellar and Virulence Type III Secretion Systems

**DOI:** 10.1128/mBio.00818-19

**Published:** 2019-06-25

**Authors:** Steven Johnson, Lucas Kuhlen, Justin C. Deme, Patrizia Abrusci, Susan M. Lea

**Affiliations:** a Sir William Dunn School of Pathology, University of Oxford, Oxford, United Kingdom; b Department of Chemistry, University of Oxford, Oxford, United Kingdom; c Central Oxford Structural Microscopy and Imaging Centre, University of Oxford, Oxford, United Kingdom; Pasteur Institute

**Keywords:** T3SS, cryo-EM, protein secretion, virulence determinants

## Abstract

Export of proteins through type III secretion systems (T3SS) is critical for motility and virulence of many major bacterial pathogens. Proteins are exported through a genetically defined export gate complex consisting of three proteins. We have recently shown at 4.2 Å that the flagellar complex of these three putative membrane proteins (FliPQR in flagellar systems, SctRST in virulence systems) assembles into an extramembrane helical assembly that likely seeds correct assembly of the rod. Here we present the structure of an equivalent complex from the *Shigella* virulence system at 3.5 Å by cryo-electron microscopy. This higher-resolution structure yields a more precise description of the structure and confirms the prediction of structural conservation in this core complex. Analysis of particle heterogeneity also suggests how the SctS/FliQ subunits sequentially assemble in the complex.

## Abstract (precis)

Although predicted on the basis of sequence conservation, the work presented here formally demonstrates that all classes of type III secretion systems, flagellar or virulence, share the same architecture at the level of the core structures. This absolute conservation of the unusual extramembrane structure of the core export gate complex now allows work to move to focusing on both mechanistic studies of type III but also on fundamental studies of how such a complex is assembled.

## INTRODUCTION

Virulence-associated type III secretion systems (T3SS), also termed injectisomes, are bacterial nanomachines that facilitate the delivery of effector proteins directly into a eukaryotic host cell cytoplasm (1, 2). Injectisomes are closely related to the T3SS at the heart of the bacterial flagellum, and both of these classes are associated with the pathogenicity of a wide range of clinically relevant bacteria (3). T3SS vary significantly, being found in both Gram-negative and Gram-positive bacteria, and with further diversity defined by the existence of extracellular and periplasmic flagella. However, at the core of all T3SS is a basal body formed by circularly symmetric protein oligomers spanning the inner membrane, from which helical flagellar filaments or injectisome needle structures project (2, 4, 5). Proteins associated with the cytoplasmic face of the basal body select proteins for export that are then transferred to a set of 5 membrane-associated proteins located at the center of the inner membrane ring (e.g., see references 6 to 13). These components (FliP, FliQ, FliR, FlhB, and FlhA in the flagellar system and SctR, SctS, SctT, SctU, and SctV in injectisomes) are collectively termed the export apparatus (EA) and are absolutely required for the translocation of substrates across the bacterial envelope (14–16).

We have recently demonstrated that a subset of these proteins assembles into a core export gate complex and reported the structure of a flagellar FliP_5_Q_4_R_1_ complex (here termed FliPQR) at 4.2 Å by cryo-electron microscopy (cryo-EM) (17). Strikingly, placement of the FliPQR structure into lower-resolution basal body structures revealed that this complex, built from three putative membrane proteins and purified from membranes when expressed in isolation of the rest of the T3SS, physiologically exists in an extramembrane location at the core of the basal body (17, 18). This positioning of the complex, in conjunction with the observation that it exhibits helical symmetry, suggested that it seeds assembly of the axial helical components that culminate in the flagellum or needle. The high level of sequence conservation within all T3SS implied that this complex would be similarly assembled in virulence T3SS. Native mass spectrometry (nMS) of purified virulence system complexes supported this, revealing a core SctR_5_T_1_ complex equivalent to FliP_5_R_1_ (17). However, the number of SctS subunits was highly variable in samples and seemed to reflect lower stability of these complexes compared with the flagellar ones (17). This decreased stability meant that determination of a structure from a nonflagellar export gate complex was not previously possible.

Here we present a re-refinement of the original FliPQR data to higher resolution (3.65 Å) and also the structure of the equivalent SctRST complex from a virulence T3SS (3.5 Å), revealing the high level of structural conservation within this core complex between flagellar and virulence T3SS. The greater fragility of the virulence complex versus the flagellar complex means that our sample contains a variety of differently assembled complexes that differ in the number of SctS (FliQ) subunits associated with the complex, supporting a model for sequential assembly of the complex coupled with extrusion from the inner membrane.

## RESULTS AND DISCUSSION

**FIG 1:**
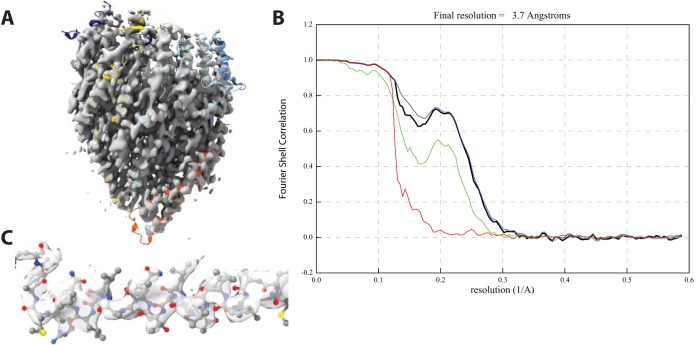
Improved resolution of *Salmonella* FliPQR reconstruction. (A) Volume obtained after re-refinement in RELION-3 with particle polishing and per-particle CTF refinement (3.65 Å, EMD-4733). (B) FSC curves. Black, FSC corrected; green, FSC unmasked maps; blue, FSC masked maps; red, FSC phase randomized. (C) Close up of region 190 to 210, chain F (FliR), to illustrate quality of density.

Re-refinement of the original Salmonella enterica serovar Typhimurium (*S. Typhimurium*) flagellar FliPQR data (17) using RELION-3 (19) yielded a significant improvement in the resolution of the density, as estimated from both Fourier shell correlation (FSC) curves and the level of detail visible in the volume (Fig. 1). We have remodeled and re-refined the coordinates into the new volume, and although there are no substantial differences, the level of confidence in these coordinates as accurately representing the biological object is clearly increased.

**FIG 2:**
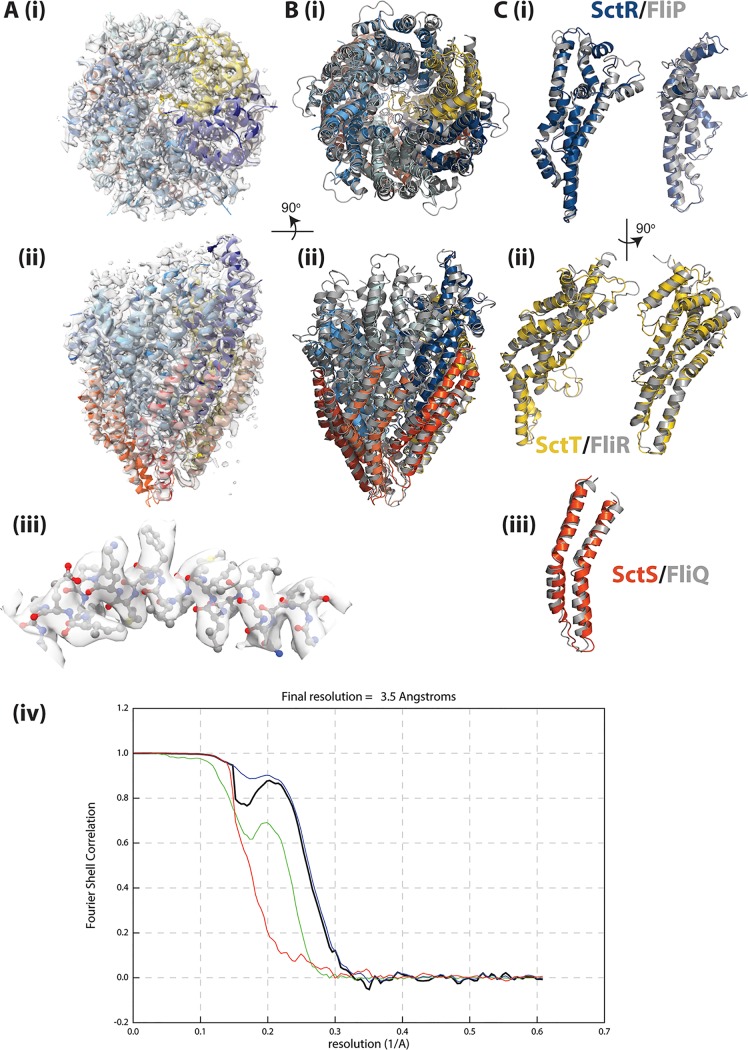
Structure of Shigella flexneri export gate (SctRST) and comparison with *Salmonella* FliPQR. (A) Different views of the coordinates within the 3.5-Å volume (EMD-4734) are shown. Panels i and ii show two views of the whole assembly related by a 90° rotation with the coordinates shown in a cartoon representation and colored (as previously defined) with five copies of SctR in shades of blue, four copies of SctS in shades of red, and with SctT in gold. Panel iii shows a close-up of a region of SctR with all atoms shown. (iv) FSC curve for reconstruction colors as in Fig. 1. (B) Overlay of Shigella flexneri SctRST (colored as in panel A) and *Salmonella* FliPQR (gray), with both shown in a cartoon representation. Views are as in panel A, subpanels i and ii. (C) Individual overlays are shown for example of each type of chain extracted from the complex, colored as in panel B.

Following our determination of the structure of the flagellar export gate complex, we expressed a variety of virulence export gate complexes using the same strategy of expression of the complete operon with a Dual-Strep tag on the C terminus of the SctT (FliR) component. Many of the systems proved fragile, with little to no SctS (FliQ) associated with the purified complexes (17), but the Shigella flexneri export gate (Spa24, Spa9, and Spa29—here referred to as SctRST) could be purified at sufficient levels to allow structure determination by single-particle cryo-EM (Fig. 2A). As previously proposed (17) based on the sequence conservation in all three components (33% identity to the *S. Typhimurium* FliPQR across the operon), the structure conclusively demonstrates the structural conservation at the core of type III secretion systems (Fig. 2B and C).

**FIG 3:**
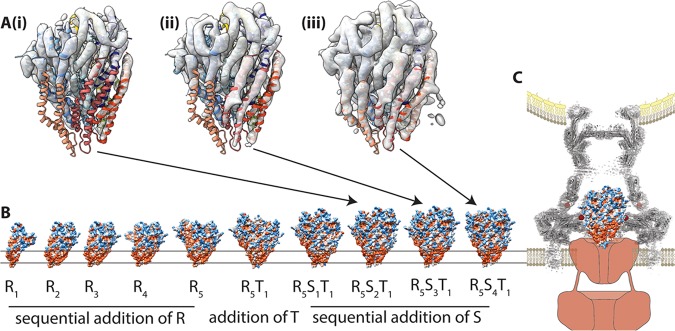
Model for assembly of the T3SS export gate. (A) 3D classification of the SctRST particles leads to volumes with different numbers of SctS subunits attached: (i) SctR_5_S_2_T_1_, (ii) SctR_5_S_3_T_1_, and (iii) the fully assembled SctR5S4T1. The full assembly is shown as a cartoon trace within each volume, with the absent SctS subunits seen to lack density at the contour level sufficient to cover the subunits present. (B) The height of the surface-exposed hydrophobic patches (orange) on the subcomplexes suggests sequential assembly in the order shown with the complex pushing out of the inner membrane into the periplasmic space. (C) Once the remaining T3SS basal body components assemble, the export gate is found above the inner membrane at the core of the basal body (gray cartoon and density [18]) with the SctV/FlhA component (red cartoon) assumed to form the channel in the inner membrane.

As suggested by the nMS analysis (17), the biggest difference between the flagellar and injectisome export gates was observed in the Q/S subunits. The highest-resolution SctRST map was generated from ∼200,000 particles and displayed density for 4 copies of SctS, but with evidence that the 4th copy, corresponding to the lowest position in the helical assembly, was substoichiometrically occupied. Further three-dimensional (3D) classification of this particle set produced a series of lower-resolution maps (Fig. 3A) highlighting the compositional heterogeneity within the SctRST sample, with evidence for SctR_5_S_2_T_1_, SctR_5_S_3_T_1_, and SctR_5_S_4_T_1_ complexes. Furthermore, the order in which the SctS copies were occupied clearly progressed down the helical assembly. This observation is in agreement with the nMS data and supports the hypothesis that the complex is assembled sequentially, with the SctS component being the last to be added.

To attempt to further understand how this ultimately extramembranous complex is assembled in the membrane, we analyzed the hydrophobic surfaces revealed when subunits are sequentially removed (Fig. 3B) from the complex. This analysis, combined with the knowledge that the complex needs to be positioned ready for extraction from the membrane to allow its incorporation into the full T3SS basal body (Fig. 3C), suggests an ordered assembly process. This would begin with sequential assembly of five copies of SctR, followed by completion of a closed structure by addition of the single copy of SctT. Creation of the R/T interface would trigger sequential addition of the four copies of SctS. This raises interesting questions of how the operon order relates to the protein complex assembly, as this implies that the order of protein assembly does not match the order of the genes within the operon.

## MATERIALS AND METHODS

### Materials.

Chemicals were obtained from Sigma-Aldrich unless otherwise specified. The detergent lauryl maltose neopentyl (LMNG) was obtained from Anatrace.

### Recombinant protein expression.

Shigella flexneri SctRST (Spa24/Spa9/Spa29) was recombinantly expressed in Escherichia coli and extracted and purified in LMNG as described previously (17). Briefly, BL21 cells transformed with plasmid pT12_Spa24929 were grown overnight in Terrific Broth supplemented with kanamycin (60 μg/ml) and rhamnose monohydrate (0.1%) at 37°C. The cells were spun down and lysed, and the membranes were pelleted by ultracentrifugation. The membranes were dissolved in 1% LMNG, and the protein was purified by StrepTrap and size exclusion chromatography.

### Cryo-EM grid preparation and single-particle data collection.

Purified SctRST in 0.01% LMNG in TBS (100 mM Tris, 150 mM NaCl, 1 mM EDTA at pH 8) was concentrated to 16.8 mg/ml and diluted to 8.4 mg/ml. Three microliters of sample was applied to glow-discharged holey carbon-coated grids (Quantifoil 300 mesh, Au R1.2/1.3), adsorbed for 5 s, blotted for 3 s at 100% humidity at 22°C, and frozen in liquid ethane using a Vitrobot Mark IV (Thermo Fisher). All EM data were collected using a Titan Krios (Thermo Fisher) operating at 300 kV. A total of 3,741 movies were collected on a K2 Summit detector (Gatan) in counting mode using a sampling of 0.822 Å/pixel (calibrated by prior determination of the structure of apo-ferrritin), with 2.4 e^−^ Å^−2^ frame^−1^ over 20 frames.

### Structure solution.

All steps in the structure solution were carried out using RELION-3 (19) unless otherwise stated. Motion correction of the movies was carried out using MotionCor2 (20), as implemented within RELION-3 (19), with dose weighting. Contrast transfer function (CTF) estimation was carried out using CTFFIND4 (21). A total of 775,073 particles were extracted in RELION-3 using boxes picked using SIMPLE (22). Reference-free 2D classification was used to select 477,653 good particles. 3D classification with 4 classes was then carried out using the *S. Typhimurium* FliPQR structure (17) low pass filtered to 40 Å as a reference. The best class (212,561 particles) was then used in 3D autorefinement, followed by Bayesian polishing and CTF parameter refinement. A final “gold standard” refinement produced the final map with a resolution of 3.5 Å after PostProcess masking and *B* factor sharpening. Model building was initially carried out using CCP4-Buccaneer, followed by manual building in Coot (23). The structure was refined using phenix.real_space_refine (24). Cryo-EM data and refinement and validation statistics are shown in Table 1.

**TABLE 1:** Cryo-EM data collection, refinement, and validation statistics

Parameter	Value for:	
	*Salmonella* FliPQR^*a*^	*Shigella* SctRST^*b*^
Data collection and processing		
Magnification, ×	165,000 (K2), 96,000 (Falcon3)	165,000 (K2)
Voltage, kV	300	300
Electron exposure, e^–^/Å^2^	47 (K2), 50 (Falcon3)	48 (K2)
Defocus range, μm	0.5–4	0.5–4
Pixel size, Å	0.85	0.822
Symmetry imposed	C1	C1
Particle images, no.		
Initial	474,625	775,073
Final	97,718	212,561
Map resolution, Å (FSC threshold)	3.65 (0.143)	3.5 (0.143)
		
Refinement		
Initial model used, PDB code	6f2d	None
Model resolution, Å (FSC threshold)	3.65 (0.143)	3.5 (0.143)
Map sharpening *B* factor, Å^2^	−80	−111
Model composition, no.		
Non-hydrogen atoms	12,541	11,416
Protein residues	1629	1,452
Ligands	0	0
*B* factors, Å^2^		
Protein	62	50
Ligand	0	0
RMSD^*c*^		
Bond lengths, Å	0.005	0.007
Bond angles, °	0.78	1.26
Validation		
MolProbity score	2.2	1.9
Clashscore	13.0	4.6
Poor rotamers, %	0.15	1.4
Ramachandran plot, %		
Favored	89.8	91.5
Allowed	9.8	8.3
Disallowed	0.4	0.2

a EMD-4733, PDB 6r69.

b EMD-4734, PDB 6r6b.

c RMSD, root mean square deviation.

### Data availability.

Volumes and coordinates have been deposited in EMDB and PDB, respectively: for *Salmonella* FliPQR under EMD-4733 and PDB-ID 6r69 and for *Shigella* SctRST under EMD-4734 and PDB 6r6b.
